# Impaired ability to modulate glomerular filtration rate in aged female sheep following fetal uninephrectomy

**DOI:** 10.1002/phy2.208

**Published:** 2014-01-28

**Authors:** Yugeesh R. Lankadeva, Reetu R. Singh, Lucinda M. Hilliard, Karen M. Moritz, Kate M. Denton

**Affiliations:** 1 Department of Physiology, Monash University, Victoria, Australia; 2 School of Biomedical Sciences, University of Queensland, St Lucia, Australia

**Keywords:** Plasma renin activity, renal blood flow, sodium excretion, tubuloglomerular feedback

## Abstract

Fetal uninephrectomy (uni‐x) results in hypertension at a later age in female than male sheep. We hypothesized that dysregulation of tubular sodium handling contributes to the reduced ability to regulate extracellular fluid (ECF) homeostasis in older females born with a congenital nephron deficit. Following renal excretory balance studies, the response to inhibition of the Na^+^K^+^2Cl^−^ cotransporter with furosemide (0.5 mg/kg bolus + 1 mg/kg per hour, i.v) or vehicle treatment was examined in conscious 5‐year‐old female uni‐x (*n* = 7) and sham (*n* = 7) sheep. Balance studies in meal‐fed sheep demonstrated that while average 24 h sodium excretion over 6 days was not different between the groups, the daily variation in sodium excretion was significantly greater in uni‐x compared to sham sheep (31 ± 4% vs. 12 ± 2%; *P* < 0.001). Basal plasma renin activity (PRA) and renal cortical cyclooxygenase‐2 (COX‐2) gene expression were lower in uni‐x sheep (both, *P* < 0.01). The increases in glomerular filtration rate (GFR) and renal blood flow observed in sham sheep in response to furosemide were significantly attenuated in uni‐x sheep (both *P*_GROUP×TREAT_ < 0.05). However, fractional sodium excretion increased by a greater extent in the uni‐x (4.4 ± 1.0%) as compared to the sham sheep (2.0 ± 0.4%; *P*_GROUP×TIME_ < 0.05) in response to furosemide. In conclusion, fetal uni‐x was associated with altered renal sodium handling and hypertension in aged females. The impaired ability to modulate PRA and GFR in the adults with a congenital nephron deficit may reduce the capacity of the kidney to respond to gains or losses in ECF to maintain a stable internal environment.

## Abstract (precis)

Losing a kidney during fetal life, results in impairments in renal sodium handling, underpinned by a reduced ability to modulate glomerular filtration rate, plasma renin activity, and tubular sodium handling in aged females. Impairments in renal autoregulatory mechanisms that buffer the kidney against fluctuations in arterial pressure, such as tubuloglomerular feedback may further contribute to progressive renal injury with advancing age.

## Introduction

A congenital nephron deficit has been strongly implicated in the development of hypertension in humans and in animal models (Kett and Denton 2010; Luyckx et al. 2013). In accord, children born with a solitary kidney that likely have a low nephron number from birth have an increased prevalence of hypertension and renal insufficiency from early adulthood (Schreuder et al. 2008; Westland et al. 2013). Females are protected from cardiovascular and renal disease compared to males, including protection from the fetal programming effects of an adverse in utero environment (Grigore et al. 2008; Rueda‐Clausen et al. 2011). Indeed, in our sheep fetal uninephrectomy (uni‐x) model, arterial pressure was increased at 6 months of age in males (Singh et al. 2009), but did not increase until 2 years of age in ovarian intact female uni‐x sheep (Singh et al. 2012a). This protection is likely due to ovarian hormones, as arterial pressure was observed to increase at 6 months of age in ovariectomized female uni‐x sheep (Moritz et al. 2002). Fetal uni‐x in sheep results in a 30% congenital deficit, without perturbations to the mother or placenta, and does not require the administration of compounds which may have deleterious effects on the development of other organs in the fetus (Douglas‐Denton et al. 2002). Moreover, the development of the permanent (metanephric) kidneys in sheep is very similar to that in humans, with both species completing nephrogenesis prior to birth (Vize et al. 1997; Moritz and Wintour 1999). Thus, sheep are a suitable model to study the long‐term repercussions of being born with a congenital nephron deficit.

Recently, we reported evidence of progressive renal impairment in female uni‐x sheep (Lankadeva et al. 2012; Singh et al. 2012a). We demonstrated that glomerular filtration rate (GFR) was reduced at 1 year of age (prior to the onset of hypertension), but that GFR had not declined further by 5 years of age in female uni‐x sheep with intact ovaries (Singh et al. 2012a). However, an age‐related decline in renal blood flow (RBF) was observed in the female uni‐x sheep, pointing to an increase in both preglomerular and postglomerular vascular resistance with age (Singh et al. 2012a). Moreover, when we physiologically challenged these 5‐year old female uni‐x sheep with a saline load, we unmasked perturbations in the regulation of GFR, tubular sodium reabsorption and the renin–angiotensin system (RAS) (Lankadeva et al. 2012). Collectively, these older female uni‐x sheep were unable to appropriately increase sodium excretion to eliminate the saline load as rapidly as age‐matched sham sheep, a response that was associated with a reduced suppression of the RAS (Lankadeva et al. 2012).

Our studies in a sheep fetal uni‐x model, and those of others using different programming models (i.e., maternal low‐protein diet, maternal glucocorticoid treatment), suggest that a reduced nephron endowment compromises the kidneys' ability to tightly regulate extracellular fluid (ECF) homeostasis, culminating in hypertension (Dagan et al. 2009; Singh et al. 2010; Moritz et al. 2011; Alwasel and Ashton 2012; Lankadeva et al. 2012). However, the mechanisms underlying the dysregulation of ECF homeostasis in these fetal programming models still remain unclear, especially in more clinically relevant older cohorts of animals. Evidence suggests that salt sensitivity of blood pressure is greater in low‐birth weight subjects (de Boer et al. 2008) and in rat offspring of mothers fed a low‐protein diet (Manning et al. 2002; Woods et al. 2004). Evidence also suggests that the Na^+^‐K^+^‐2Cl^−^ cotransporter (NKCC2) in the thick ascending limb of the loop of Henle may be an important determinant of this increase in salt sensitivity (Manning et al. 2002; Alwasel and Ashton 2012). Furthermore, aging females have greater blood pressure sensitivity to salt than males (He et al. 2009).

It was our hypothesis that dysregulation of tubular handling of sodium contributes to the reduced ability to regulate ECF homeostasis in older female sheep born with a congenital nephron deficit. Loop diuretics, such as furosemide, induce their potent diuretic effects not only by inhibiting the NKCC2 cotransporters in the thick ascending limb of the Henle's loop, but also by inhibiting the NKCC2 cotransporters in the macula densa, thus blocking tubuloglomerular feedback (TGF) (Wright and Schnermann 1974; Vallon 2003). The aim of this study was to assess renal handling of sodium in conscious female uni‐x and sham sheep at 5 years of age by examining sodium and water balance over a 6‐day period and the renal response to inhibition of the NKCC2 cotransporter with furosemide. Additionally, given, the known role of prostanoids in the modulation of renal function, particularly TGF, and evidence that this system may be impaired in models of low nephron endowment (Baserga et al. 2007; Brennan et al. 2008), we also examined the renal expression of cyclooxygenase‐1 (COX‐1) and cyclooxygenase‐2 (COX‐2) in these sheep.

## Methods

### Animals

Merino ewes carrying fetuses of known gestational age underwent uni‐x or sham surgery at 100 days postconception, as previously described (uni‐x, *n* = 7; sham *n* = 7) (Moritz et al. 1999). Only female fetuses were selected for use in this study. After birth, lambs remained with their mothers on pasture until weaned at 18 weeks of age. At 5 months of age, the right carotid artery was exteriorized into a skin fold to form a carotid arterial loop, as previously described (Dodic et al. 1998). Other studies in this cohort of animals have been previously reported (Lankadeva et al. 2012; Singh et al. 2012a). All experiments were approved by the Monash University, School of Biomedical Sciences Animal Ethics Committee, and were carried out in agreement with the guidelines of the National Health and Medical Research Council of Australia.

### Protocol 1: 24‐h sodium and water balance over a 6‐day period

The uni‐x and sham sheep were brought into the laboratory at 5‐years of age, placed into individual metabolic cages, and acclimatized to a diet of lucerne chaff (1 kg) and 5 L of water presented at 1700 h each day for a week. Twenty‐four hour food and water intake and urine output were measured over a 6‐day period. Urine samples were collected and analyzed for sodium concentration (RapidChem 744 Electrolyte analyser, Siemans Healthcare Diagnostics Inc, Deerfield, IL). A period of 1 week was allowed before the next protocol was performed, during which minor preparatory surgery to insert catheters was performed.

### Protocol 2: basal response to furosemide infusion in conscious female uni‐x sheep

The sheep were instrumented with a tygon catheter placed into the carotid loop for measurement of arterial pressure, and the left and right jugular veins were catheterized for infusion purposes 3 days prior to the study, as previously described (Lankadeva et al. 2012). Mean arterial pressure (MAP) and heart rate (HR) were recorded continuously, as described previously (Moritz et al. 2002). On the day prior to study, a removable Foley catheter was inserted into the bladder to enable urine collection and measurement of renal function. GFR was determined via the clearance of ^51^chromium‐ethylenediamine‐tetra‐acetic acid (^51^Cr EDTA, 15 *μ*Ci bolus + 15 *μ*Ci/h, i.v) and effective renal plasma flow (ERPF) and hence RBF (ERPF/(1‐hematocrit)) via the clearance of para‐aminohippuric acid (PAH) as previously reported (Lankadeva et al. 2012). Filtration fraction (FF), urine flow (UF), urinary sodium excretion (U_Na+_V), filtered load of sodium (FLNa), and fractional sodium excretion (FENa) were calculated as previously reported (Lankadeva et al. 2012). Plasma and urine ^51^Cr EDTA concentrations were measured using a gamma counter and PAH was measured using a rapid microplate assay, as previously described (Agarwal 2002). Plasma and urinary sodium concentrations were measured using a RapidChem 744 Electrolyte analyser and plasma renin activity (PRA) was measured by radio‐immunoassay (Prosearch International, Melbourne, Australia).

Measurements of arterial pressure and renal function were made during a control period, consisting of two 30‐min urine collections with arterial blood samples (5 mL) collected at the midpoint of each urine collection. Additional arterial blood samples (5 mL) were collected into a chilled tube containing EDTA for the measurement of PRA. These measurements were pooled to provide a single control value (control). Then an infusion of furosemide (0.5 mg/kg bolus + 1 mg/kg per hour, i.v.; Sigma‐Aldrich, St Louis, MO) or vehicle (0.9% isotonic saline) commenced. Following a 30‐min equilibration period, a 30‐min urine sample (treatment period) was collected, with arterial blood samples collected at the midpoint. Each sheep received the vehicle and furosemide infusion in separate studies, 3 days apart.

### Gene expression

Three weeks following the completion of all experiments, animals were humanely euthanized (pentobarbitone, Lethabarb^®^, Virbac Pty. Ltd, Milpera, NSW, Australia). A 0.5‐cm slice was taken from the right kidney, in transverse plane, and further subdivided into cortical and medullary sections and snap frozen in liquid nitrogen. These sections were then cut into smaller pieces containing equal proportions of cortex and medulla, weighed and homogenized to extract RNA for determining gene expression of the COX‐1 and COX‐2 using SYBR green chemistry on an Eppendorf RealPlex Cycler real‐time machine (Perkin‐Elmer Applied Biosystems, Foster City, CA). The forward primer sequence used for COX‐1 was 5′ ATG AGT ACC GCA AGA GGT TTG G 3′, the reverse primer sequence was 5′ ACG TGG AAG GAG ACA TAG G 3′. The forward primer sequence for COX‐2 was 5′ CAG AGC TCT TCC TCC TGT GC 3′; the reverse primer was 5′ CAA AAG GCG ACG GTT ATG C 3′, respectively. A comparative cycle of *C*_*T*_ (threshold fluorescence) method using 18S as the housekeeping gene was used as previously described (Singh et al. 2010).

### Statistical analysis

All values are expressed as mean ± standard error of the mean (SEM). All renal variables were corrected for body weight (kg BW). Protocol 1: these data were assessed using repeated measures analysis of variance (ANOVA) with factors group (*P*_GROUP_: uni‐x or sham), time (*P*_TIME_), and their interaction (*P*_GROUP×TIME_). In addition, the coefficient of variation of the data was calculated across the collection days and compared between the groups via an unpaired *t*‐test. Protocol 2: the change in each variable in response to treatment (furosemide or vehicle) as compared to the respective control period were analyzed using repeated measures ANOVA with factors group (*P*_GROUP_: sham or uni‐x), time (*P*_TIME_), and their interaction (*P*_GROUP×TIME_). Gene expression data were analyzed by unpaired *t*‐test with Bonferroni correction to conservatively adjust for multiple comparisons. Statistical analysis was performed using GraphPAD PRISM 6.0 (Graphpad Sofware Inc, La Jolla, CA), where two‐sided *P* ≤ 0.05 was considered statistically significant.

## Results

### Protocol 1: 24‐h sodium and water balance over a 6‐day period

**Figure 1.**
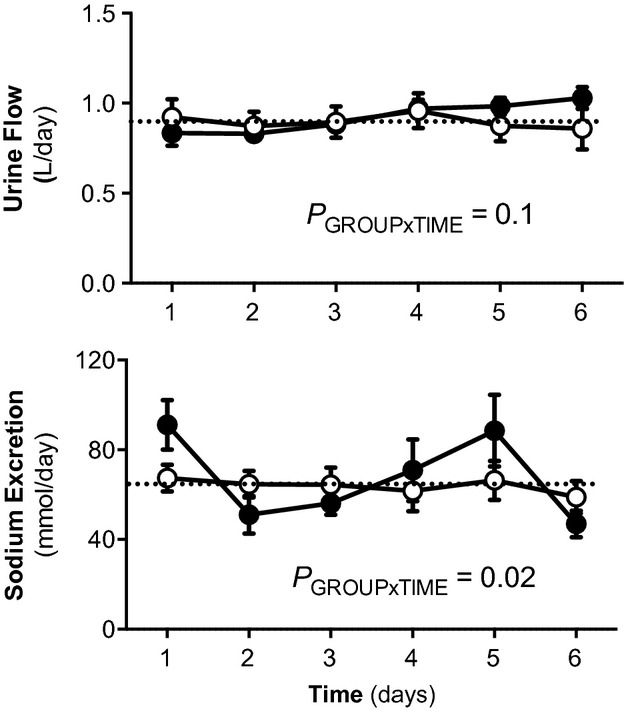
Daily (24‐h) urine output and urinary sodium excretion in sham and uni‐x sheep at 5 years of age. Variables (mean ± SEM) measured for 6 days in 5‐year‐old sham (*n* = 7; open circles) and uni‐x (closed circles; *n* = 7) meal‐fed female sheep. The dotted line represents the average value for that variable in the sham sheep. *P* value represents the interaction term from a repeated measures ANOVA with factors group (sham or uni‐x) and time.

Daily urine output was not significantly different between the 5‐year‐old female sham and uni‐x sheep across the 6‐day period (Fig. 1). However, while sodium excretion was relatively constant each day in the sham sheep, sodium excretion varied significantly more widely each day in the uni‐x sheep. Analysis of the coefficient of variation for sodium excretion demonstrated that sodium excretion varied by 31 ± 4% each day in the uni‐x sheep as compared to 12 ± 2% in the sham sheep (*P*_GROUP×TIME_ < 0.01; Fig. 1). There was no difference in BW (sham, 61 ± 2 kg; uni‐x, 58 ± 2 kg) or total kidney weight (sham, 110 ± 7 g; uni‐x 103 ± 6 g) between sham and uni‐x sheep at 5 years of age. Daily food intake (meal‐fed) and water intake was similar in both groups over the 6 days of balance measurements (sham, 2.79 ± 0.05 L; uni‐x, 2.86 ± 1.1 L; *P* = 0.6).

### Protocol 2: basal response to furosemide infusion in conscious female uni‐x sheep

**Figure 2.**
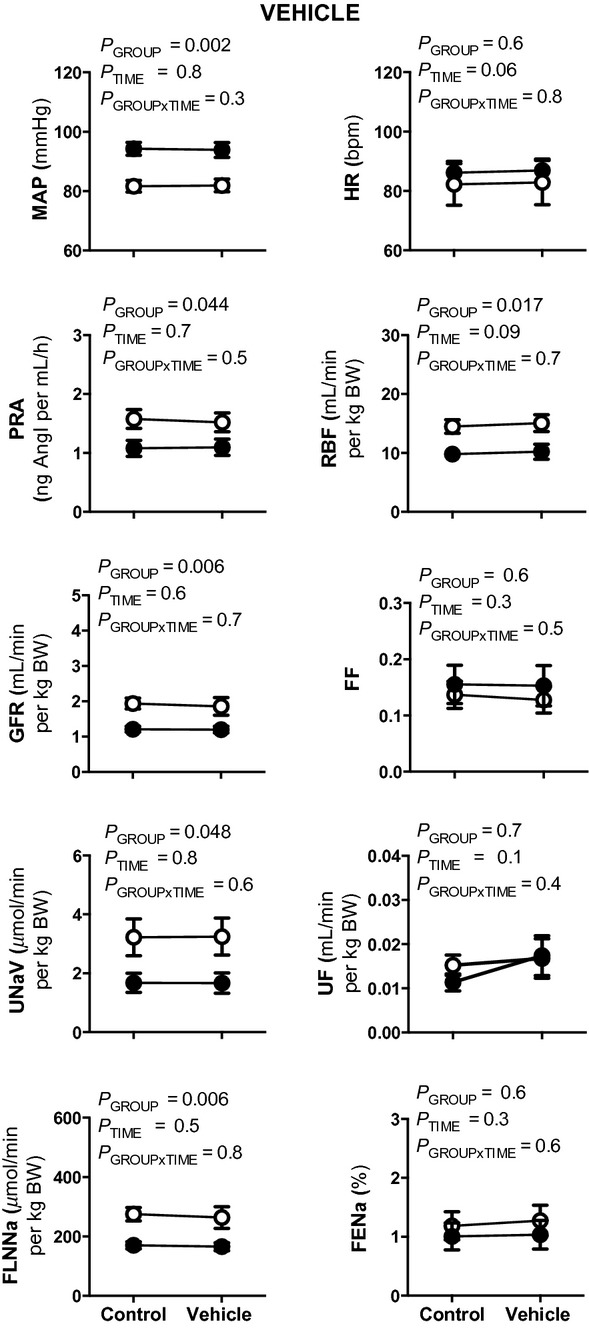
Effect of vehicle treatment on cardiovascular and renal variables in conscious 5‐year‐old sham and uni‐x sheep. Mean arterial pressure (MAP), heart rate (HR), plasma renin activity (PRA), effective renal blood flow (RBF), glomerular filtration rate (GFR), filtration fraction (FF), urine flow (UF), urinary sodium excretion (U_Na+_V), filtered load of sodium (FLNa), and fractional sodium excretion (FENa) measured before and during vehicle treatment in sham (open circles, *n* = 7) and uni‐x (closed circles, *n* = 7) sheep. Values are represented as mean ± SEM. All renal variables are presented as absolute values corrected for gram of total body weight (per g BW). *P* values represent the results of repeated measures ANOVA, with factors group (sham or uni‐x), time (before or after vehicle) and their interaction.

Basal MAP was ~12 mmHg greater and basal U_Na+_V (~30%), GFR (~37%), RBF (~33%), and the FLNa (~30%) were all lower in the uni‐x as compared to the sham group (All *P*_GROUP_ < 0.05; Fig. 2). Basal PRA was ~35% lower in the uni‐x as compared to the sham sheep (*P*_GROUP_ = 0.044; Fig. 2). Basal HR, UF, FF, and FENa were not significantly different between the uni‐x and sham groups (Fig. 2). Vehicle treatment did not significantly affect any measured variable (Fig. 2).

**Figure 3.**
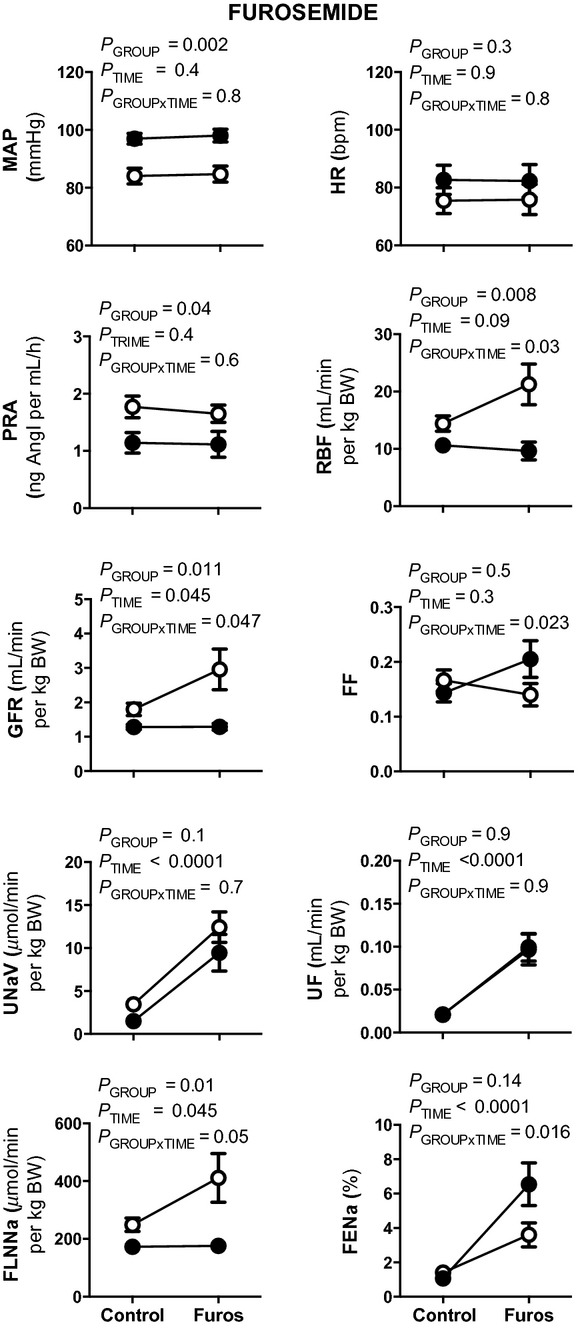
Effect of furosemide treatment on cardiovascular and renal variables in conscious 5‐year‐old sham and uni‐x sheep. Mean arterial pressure (MAP), heart rate (HR), plasma renin activity (PRA), effective renal blood flow (RBF), glomerular filtration rate (GFR), filtration fraction (FF), urine flow (UF), urinary sodium excretion (U_Na+_V), filtered load of sodium (FLNa), and fractional sodium excretion (FENa) measured before and during furosemide treatment in sham (open circles, *n* = 7) and uni‐x (closed circles, *n* = 7) sheep. Values are represented as mean ± SEM. All renal variables are presented as absolute values corrected for gram of total body weight (per g BW). *P* values represent the results of repeated measures ANOVA, with factors group (sham or uni‐x), time (before or after furosemide) and their interaction.

Furosemide infusion did not significantly alter MAP, HR, or PRA in either sham or uni‐x groups (Fig. 3). However, GFR and RBF were differentially affected by furosemide infusion in the two groups. In response to furosemide, GFR and RBF increased by 46 ± 17% and 60 ± 25%, respectively, in the sham group, whilst in comparison the increase in GFR was only 3 ± 11% in the uni‐x group (*P*_GROUP×TIME_ = 0.047), coupled with a 11 ± 13% reduction in RBF (*P*_GROUP×TIME_ = 0.03; Fig. 2). The change in FF in response to furosemide was significantly different between the groups, with FF decreasing in the sham but increasing in the uni‐x sheep (Fig. 3; *P*_GROUP×TIME_ = 0.023).

UF (~440%; *P*_GROUP×TIME_ = 0.9; Fig. 3) and U_Na+_V (~500%; *P*_GROUP×TIME_ = 0.7; Fig. 3) increased by a similar extent in both groups following furosemide infusion. However, whilst the FLNa increased in response to furosemide infusion in the sham sheep, this response was significantly attenuated in the uni‐x sheep (sham, 60 ± 24%; uni‐x, 4 ± 10%; *P*_GROUP×TIME_ = 0.045; Fig. 3). FENa increased significantly in both groups in response to furosemide infusion, however, the response was greater in the uni‐x as compared to the sham sheep (*P*_GROUP×TIME_ = 0.016; Fig. 3).

### COX‐1 and COX‐2 mRNA expression

**Figure 4.**
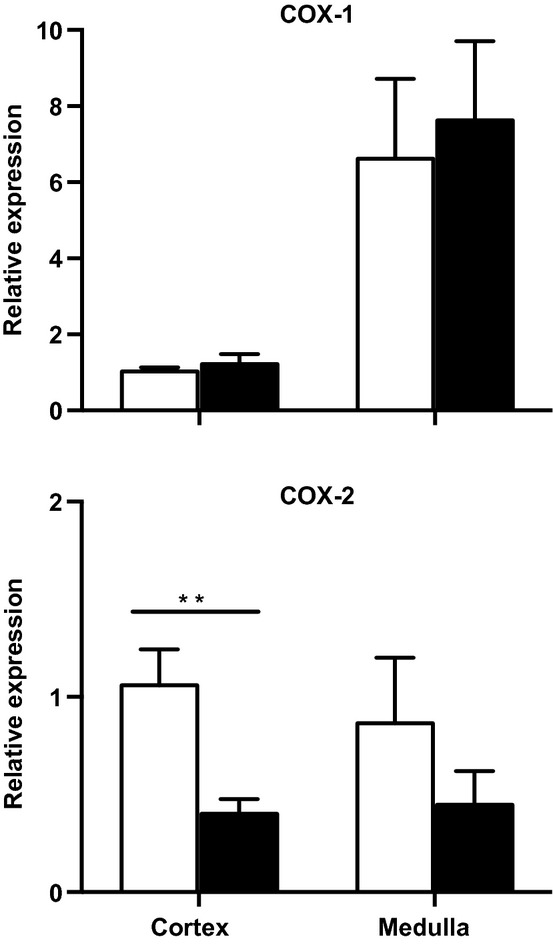
Effect of fetal uni‐x on renal cortical and medullary mRNA expression of COX‐1 and COX‐2 in female sheep at 5 years of age. All gene expression was normalized to a calibrator (sham cortex) with 18S as the housekeeping gene in sham (*n* = 5; open bars) and uni‐x (*n* = 5; closed bars) sheep. Values are represented as mean ± SEM. *P* values represent results from a two‐tailed unpaired Student's *t*‐test. **P < 0.01.

At 5 years of age, the uni‐x sheep had a significantly lower expression of COX‐2 mRNA in the renal cortex compared to the sham animals (*P* = 0.01; Fig. 4). However, there was no significant difference in the relative expression of COX‐2 within the renal medulla between treatment groups (Fig. 4). While the expression of COX‐1 was greater in the renal medulla as compared to the cortex in both sham and uni‐x sheep (*P* = 0.004; Fig. 4), there was no difference in COX‐1 expression in the two kidney zones between the treatment groups.

## Discussion

The main finding of this study was that the normal tight matching of sodium output to intake was observed to oscillate by a significantly greater degree in aged female sheep born with a solitary kidney. In meal‐fed normotensive sham sheep sodium excretion varied by ~10% each day, whereas sodium excretion fluctuated by as much as 30% each day in the 5‐year‐old female uni‐x sheep. Furthermore, we observed that while sodium excretion increased by a similar extent in both the sham and uni‐x groups in response to furosemide infusion, the mechanisms driving the increase were different. This indicates that regulation of renal handling of sodium via the NKCC2 cotransporter was altered in 5‐year‐old female uni‐x sheep.

It is well‐recognized that young females are protected from renal and cardiovascular disease and that with advancing age this protection is lost (Reckelhoff 2008). Previously, in this cohort of female sheep that underwent unilateral nephrectomy at 100 days of gestation (term = 150 days), we reported a decrease in GFR at 1 year of age prior to an increase in arterial pressure (Singh et al. 2012a). In the current study, we demonstrate that these same female uni‐x sheep at 5 years of age have significant renal impairments and sustained hypertension as compared to age‐matched sham sheep. In contrast, we have previously shown that male uni‐x sheep develop renal dysfunction and hypertension at 6 months of age (Singh et al. 2009). Thus, loss of a kidney during fetal life results in impaired renal function and hypertension in adulthood, albeit at a more advanced age in females. These findings are in accord with other animal models of reduced nephron endowment (Ozaki et al. 2001; Alexander 2003; Woods et al. 2005) and studies in patients with unilateral renal agenesis (Parikh et al. 2002). Clearly, it is important to understand the mechanisms underpinning this reduction in renal function associated with a reduced renal mass at birth, if we are to slow further loss of renal function and prevent cardiovascular disease with the advancement of age. Our study sheds some light upon this question.

The kidney, by maintaining an appropriate balance between sodium and fluid intake and renal excretion, keeps the ECF environment remarkably stable and plays a dominant role in the long‐term regulation of arterial pressure (Bie and Damkjaer 2010). The present study provides evidence to suggest that the dynamic regulation of sodium homeostasis is perturbed in female uni‐x sheep at 5 years of age. In the meal‐fed sham animals as expected daily sodium excretion was held relatively constant over a period of 6 days, whereas in the uni‐x sheep sodium excretion undershot and overshot the average sodium excretion by as much as 30% each day. Similar findings were previously observed in male uni‐x sheep, but earlier, at 6 months of age (Singh et al. 2010). At odds with this finding was the observation that during the clearance studies (urine collected via bladder catheter vs. voluntary voiding in the 24 h studies) basal sodium excretion was significantly lower in the uni‐x sheep, this requires some explanation. Firstly, these animals received a constant sodium intake with free access to water throughout these studies. During the 6‐day 24 h collections, we observed significant variation in sodium excretion in the uni‐x animals. Therefore, it is possible that during the clearance study sodium excretion was at the lowest end of that oscillation. However, this seems too fortuitous and we suggest that the stress associated with this protocol may have driven the decrease in sodium excretion and speculate that retained ECF would be excreted in the following days. Certainly, increased stress responses and increased activity of the renal sympathetic nerves have been implicated in the fetal programming of hypertension (Kett and Denton 2010). Finally, it is possible loss of sodium via the gastrointestinal tract, saliva and the fact that sheep can sequester fluid in the stomach, may account for differences in sodium excretion. However, taken together our findings suggest that the renal mechanisms regulating sodium excretion are impaired in the solitary kidney of aged female uni‐x sheep, which likely contributes to the observed increase in arterial pressure.

To investigate this finding further, the contribution of the NCCK2 cotransporter to renal sodium excretion was examined in 5‐year‐old female uni‐x sheep. Previously, the NCCK2 cotransporter has been demonstrated to be increased in animal models with a congenital reduction in nephron number (Manning et al. 2002; Dagan et al. 2009; Alwasel and Ashton 2012). Furosemide is a potent diuretic, which acts to inhibit NKCC2 cotransporters in the thick ascending limb of Henle's loop and the macula densa. Thus, administration of furosemide leads to a decrease in fluid and electrolyte reabsorption in the thick ascending limb and blocks TGF, consequently leading to diuresis and natriuresis (Wright and Schnermann 1974; Duchin et al. 1977; Tucker and Blantz 1984). In sham sheep, there was a marked diuresis and natriuresis that was associated with an increase in RBF and GFR in response to furosemide infusion. The increase in RBF was relatively greater than the rise in GFR, resulting in a reduction in FF, which is in agreement with our previous reports in 6‐month‐old male sham sheep (Singh et al. 2012b). This finding suggests that the vasodilatation in response to furosemide was predominantly preglomerular, and the observed increase in RBF and GFR was the result of the removal of the tonic constrictor effect on the afferent arterioles exerted by TGF (Kurokawa 1998; Vallon 2003; Blantz et al. 2007). Therefore, the diuresis and natriuresis observed in sham sheep in response to furosemide can be attributed to both an increase in the filtered load, as well as a reduction in tubular sodium reabsorption in response to the diuretic, in agreement with previous studies (Christensen and Petersen 1988; Singh et al. 2012b).

In contrast, the factors driving natriuresis in the uni‐x animals in the presence of furosemide were different to that of sham sheep, suggesting alterations in the tubular handling of sodium within the uni‐x kidney. In the uni‐x sheep, the increase in sodium excretion was solely due to a reduction in tubular reabsorption of sodium as the FLNa did not increase. Thus, it can be inferred that as sodium excretion increased by a similar degree in response to furosemide infusion in the uni‐x as compared to the sham animals that the contribution of the NKCC2 cotransporter to sodium reabsorption in the kidney was enhanced in the uni‐x animals. Indeed, we and others have reported considerable renal tubular hypertrophy (Hayslett et al. 1968; Pollock et al. 1992; Singh et al. 2012a) and an upregulation of tubular sodium transporters following unilateral nephrectomy (Girardi et al. 2002; Singh et al. 2010; Lankadeva et al. 2012). A limitation of the present study was the inability to determine expression levels of the NKCC2 cotransporter due to the gene sequence being unknown and the unavailability of antibodies specific for sheep. Thus, whether the increased contribution of the NKCC2 cotransporter to sodium excretion is due to an increase in number or activity of these transporters requires further investigation.

Unlike the sham animals, RBF and GFR did not increase in the uni‐x animals in response to furosemide and thus an increase in the FLNa did not contribute to the increase in sodium excretion. In the sham animals, the increase in GFR and RBF was likely due to inhibition of NKCC2 cotransporters on the macula densa leading to removal of tonic TGF activity. Therefore, the attenuated GFR and RBF response in uni‐x sheep to furosemide suggest that TGF is impaired and that this contributes to the loss of tight control of sodium excretion in animals born with a congenital renal mass reduction. A rightward resetting of TGF to function at a higher single nephron GFR and solute delivery to the distal tubules has been shown to occur acutely in adult rats following nephrectomy (Diezi et al. 1976; Aperia et al. 1978; Blantz et al. 1991). However, if the enhanced delivery of solutes to the macula densa persists for a prolonged period of time, the TGF mechanism adapts by reducing sensitivity to changes in distal volume delivery, a response that is usually associated with a reduction in renin secretion (Muller‐Suur et al. 1980; Thomson et al. 1996; Schnermann et al. 1998). It is possible that this is due to a downregulation of the NKCC2 cotransporter in the macula densa cells, but given our evidence that sodium reabsorption via the NKCC2 cotransporter is enhanced in the uni‐x sheep, we think this is unlikely and suggest that downstream components of the TGF pathway are suppressed. Alternatively, studies in isolated blood vessels have demonstrated the potential for furosemide to cause direct vasodilatation via inhibition of the NKCC1 transporter (Dormans et al. 1996; Oppermann et al. 2007). Therefore, part of the renal response to furosemide in sham sheep might be explained by NKCC1 inhibition leading to direct vasodilatation. In turn, downregulation of the NKCC1 transporter on the renal vasculature in the uni‐x kidney might account in part for the differential RBF response to furosemide.

The RAS is a key modulator of TGF. Recently, we reported that renal tissue levels of renin and angiotensin II and renal gene expression for angiotensin II Type 1 receptor gene expression were reduced in female uni‐x sheep at 5 years of age (Singh et al. 2013). This was supported by the functional demonstration that the renal response to angiotensin II infusion was significantly attenuated in the uni‐x sheep (Singh et al. 2013). Given the known role of angiotensin II in modulating TGF (Schnermann and Briggs 2008), this suppression of the renal RAS may be involved in the impairment of TGF in uni‐x sheep. Furthermore, prostaglandin generation via COX‐2, which is highly expressed in the macula densa, also modulates TGF and renin release (Persson et al. 1984; Deng et al. 2004; Green et al. 2012). In the present study, we demonstrated that uni‐x sheep had significantly lower renal cortical COX‐2 expression in association with lower plasma renin levels. Thus, the downregulation of renal cortical COX‐2 in uni‐x sheep may also contribute to the impairment of TGF in uni‐x sheep. A limitation of the current study is that due to the lack of specific antibodies for COX‐2 in the sheep, the downregulation of COX‐2 at the protein level could not be confirmed. It may be necessary to perform future studies in rodents, in which antibodies are available, to address these issues directly. Indeed, previous studies have suggested a role for the suppression of prostanoids in models of low nephron endowment (Baserga et al. 2007; Brennan et al. 2008). The indirect assessment of the role of TGF via NKCC2 inhibition at the level of the whole kidney is a limitation of this study. However, the data does strongly suggest that TGF is impaired in 5‐year‐old female sheep that underwent fetal uni‐x. Therefore, future studies are warranted to directly assess TGF at the level of the single nephron via renal micropuncture, in order to gain more mechanistic insight into the perturbations associated with the regulation of renal function in models of low nephron endowment.

## Conclusion

In conclusion, while younger females are protected from the deleterious effects of a congenital nephron deficit, fetal uni‐x was associated with altered renal handling of sodium and hypertension in aged females. The lack of change in renal hemodynamics in response to furosemide, in combination with the reduced renal cortical COX‐2 expression and lower PRA suggests that TGF is impaired in aged females following the fetal loss of a kidney. Thus, the impaired ability to modulate PRA and GFR in the adult following a congenital renal mass reduction may reduce the capacity of the remaining kidney to respond to gains or losses in ECF in order to maintain a stable internal environment.

## Acknowledgments

The authors would like to thank Alex Satragno, Alan McDonald and Andrew Jefferies for assistance in surgical preparation of the animals.

## Conflict of Interest

None declared.

## References

[b1] Agarwal R. 2002 Rapid microplate method for PAH estimation. Am. J. Physiol. Renal Physiol.; 283:F236-F241 12110506 10.1152/ajprenal.00336.2001

[b2] Alexander B. T. 2003 Placental insufficiency leads to development of hypertension in growth‐restricted offspring. Hypertension; 41:457-462 12623943 10.1161/01.HYP.0000053448.95913.3D

[b3] Alwasel S. Ashton N. 2012 Segmental sodium reabsorption by the renal tubule in prenatally programmed hypertension in the rat. Pediatr. Nephrol.; 27:285-293 21863227 10.1007/s00467-011-1976-9

[b4] Aperia A. Broberger O. Wilton P. 1978 Renal functional adaptation in the remnant kidney in patients with renal agenesis and in patients nephrectomized in childhood. Acta Paediatr.; 67:611-615 10.1111/j.1651-2227.1978.tb17811.x 696307

[b5] Baserga M. Hale M. A. Wang Z. M. Yu X. Callaway C. W. McKnight R. A. 2007 Uteroplacental insufficiency alters nephrogenesis and downregulates cyclooxygenase‐2 expression in a model of IUGR with adult‐onset hypertension. Am. J. Physiol. Regul. Integr. Comp. Physiol.; 292:R1943-R1955 17272666 10.1152/ajpregu.00558.2006

[b6] Bie P. Damkjaer M. 2010 Renin secretion and total body sodium: pathways of integrative control. Clin. Exp. Pharmacol. Physiol.; 37:e34-e42 19843096 10.1111/j.1440-1681.2009.05316.x

[b7] Blantz R. C. Peterson O. W. Thomson S. C. 1991 Tubuloglomerular feedback responses to acute contralateral nephrectomy. Am. J. Physiol. Renal Physiol.; 260:F749-F756 10.1152/ajprenal.1991.260.5.F749 2035659

[b8] Blantz R. C. Deng A. Miracle C. M. Thomson S. C. 2007 Regulation of kidney function and metabolism: a question of supply and demand. Trans. Am. Clin. Climatol. Assoc.; 118:23-43 18528487 PMC1863590

[b9] de Boer M. P. IJzerman R. G. de Jongh R. T. Eringa E. C. Stehouwer C. D. A. Smulders Y. M. 2008 Birth weight relates to salt sensitivity of blood pressure in healthy adults. Hypertension; 51:928-932 18287343 10.1161/HYPERTENSIONAHA.107.101881

[b10] Brennan K. A. Kaufman S. Reynolds S. W. McCook B. T. Kan G. Christiaens I. 2008 Differential effects of maternal nutrient restriction through pregnancy on kidney development and later blood pressure control in the resulting offspring. Am. J. Physiol. Regul. Integr. Comp. Physiol.; 295:R197-R205 18480243 10.1152/ajpregu.00741.2007 PMC2494803

[b11] Christensen S. Petersen J. S. 1988 Effects of furosemide on renal haemodynamics and proximal tubular sodium reabsorption in conscious rats. Br. J. Pharmacol.; 95:353-360 3228668 10.1111/j.1476-5381.1988.tb11653.x PMC1854190

[b12] Dagan A. Gattineni J. Cook V. Baum M. 2009 Prenatal programming of rat thick ascending limb chloride transport by low‐protein diet and dexamethasone. Am. J. Physiol. Regul. Integr. Comp. Physiol.; 297:R93-R99 19403862 10.1152/ajpregu.91006.2008 PMC2711704

[b13] Deng A. Wead L. Blantz R. 2004 Temporal adaptation of tubuloglomerular feedback: effects of COX‐2. Kidney Int.; 66:2348-2353 15569325 10.1111/j.1523-1755.2004.66033.x

[b14] Diezi J. Michoud P. Grandchamp A. Giebisch G. 1976 Effects of nephrectomy on renal salt and water transport in the remaining kidney. Kidney Int.; 10:450-462 1011539 10.1038/ki.1976.132

[b15] Dodic M. May C. N. Wintour E. M. Coghlan J. P. 1998 An early prenatal exposure to excess glucocorticoid leads to hypertensive offspring in sheep. Clin. Sci.; 94:149-155 9536923 10.1042/cs0940149

[b16] Dormans T. P. J. Pickkers P. Russel F. G. M. Smits P. 1996 Vascular effects of loop diuretics. Cardiovasc. Res.; 32:988-997 9015400 10.1016/s0008-6363(96)00134-4

[b17] Douglas‐Denton R. Moritz K. M. Bertram J. F. Wintour E. M. 2002 Compensatory renal growth after unilateral nephrectomy in the ovine fetus. J. Am. Soc. Nephrol.; 13:406-410 11805169 10.1681/ASN.V132406

[b18] Duchin K. L. Peterson L. N. Burke T. J. 1977 Effect of furosemide on renal autoregulation. Kidney Int.; 12:379-386 609187 10.1038/ki.1977.128

[b19] Girardi A. C. C. Rocha R. O. Britto L. R. G. Reboucas N. A. 2002 Upregulation of NHE3 is associated with compensatory cell growth response in young uninephrectomized rats. Am. J. Physiol. Renal Physiol.; 283:F1296-F1303 12388404 10.1152/ajprenal.00010.2002

[b20] Green T. Gonzalez A. Mitchell K. Navar L. 2012 The complex interplay between cyclooxygenase‐2 and angiotensin II in regulating kidney function. Curr. Opin. Nephrol. Hypertens.; 21:7-14 22080858 10.1097/MNH.0b013e32834d9d75 PMC3281505

[b21] Grigore D. Ojeda N. B. Alexander B. T. 2008 Sex differences in the fetal programming of hypertension. Gend. Med.; 5Suppl. A:S121-S132 18395678 10.1016/j.genm.2008.03.012 PMC2657344

[b22] Hayslett J. Kashgarian M. Epstein F. 1968 Functional correlates of compensatory renal hypertrophy. J. Clin. Invest.; 47:774-799 5641618 10.1172/JCI105772 PMC297228

[b23] He J. Gu Chen J. Jaquish C. E. Rao D. C. Hixson J. E. 2009 Gender difference in blood pressure responses to dietary sodium intervention in the GenSalt study. J. Hypertens.; 27:48-5410.1097/HJH.0b013e328316bb87 19145767 10.1097/hjh.0b013e328316bb87 PMC2882679

[b24] Kett M. M. Denton K. M. 2010 Renal programming: cause for concern? Am. J. Physiol. Regul. Integr. Comp. Physiol.; 300:R791-R803 21191002 10.1152/ajpregu.00791.2010

[b25] Kurokawa K. 1998 Tubuloglomerular feedback: its physiological and pathophysiological significance. Kidney Int.; 54:S71-S74 10.1046/j.1523-1755.1998.06714.x 9736257

[b26] Lankadeva Y. R. Singh R. R. Hilliard L. M. Moritz K. M. Denton K. M. 2012 Blunted sodium excretion in response to a saline load in 5 year old female sheep following fetal uninephrectomy. PLoS One; 7:e47528 23077628 10.1371/journal.pone.0047528 PMC3471853

[b27] Luyckx V. A. Bertram J. F. Brenner B. M. Fall C. Hoy W. E. Ozanne S. E. 2013 Effect of fetal and child health on kidney development and long‐term risk of hypertension and kidney disease. Lancet; 382:273-283 23727166 10.1016/S0140-6736(13)60311-6

[b28] Manning J. Beutler K. Knepper M. A. Vehaskari V. M. 2002 Upregulation of renal BSC1 and TSC in prenatally programmed hypertension. Am. J. Physiol. Renal Physiol.; 283:F202-F206 12060603 10.1152/ajprenal.00358.2001

[b29] Moritz K. M. Wintour E. M. 1999 Functional development of the meso‐ and metanephros. Pediatr. Nephrol.; 13:171-178 10229008 10.1007/s004670050587

[b30] Moritz K. M. Macris M. Talbo G. Wintour E. M. 1999 Foetal fluid balance and hormone status following nephrectomy in the foetal sheep. Clin. Exp. Pharmacol. Physiol.; 26:857-864 10561805 10.1046/j.1440-1681.1999.03155.x

[b31] Moritz K. M. Wintour E. M. Dodic M. 2002 Fetal uninephrectomy leads to postnatal hypertension and compromised renal function. Hypertension; 39:1071-1076 12052844 10.1161/01.hyp.0000019131.77075.54

[b32] Moritz K. M. De Matteo R. Dodic M. Jefferies A. J. Arena D. Wintour E. M. 2011 Prenatal glucocorticoid exposure in the sheep alters renal development in utero: implications for adult renal function and blood pressure control. Am. J. Physiol. Regul. Integr. Comp. Physiol.; 301:R500-R509 21593424 10.1152/ajpregu.00818.2010

[b33] Muller‐Suur R. Norlen B. J. Erik A. Persson G. Muller‐Suur C. Forsmark B. 1980 Resetting of tubuloglomerular feedback in rat kidneys after unilateral nephrectomy. Kidney Int.; 18:48-57 7012420 10.1038/ki.1980.109

[b34] Oppermann M. Hansen P. B. Castrop H. Schnermann J 2007 Vasodilatation of afferent arterioles and paradoxical increase of renal vascular resistance by furosemide in mice. Am. J. Physiol. Renal Physiol.; 293:F279-F287 17494095 10.1152/ajprenal.00073.2007

[b35] Ozaki T. Nishina H. Hanson M. A. Poston L. 2001 Dietary restriction in pregnant rats causes gender‐related hypertension and vascular dysfunction in offspring. J. Physiol.; 530:141-152 11136866 10.1111/j.1469-7793.2001.0141m.x PMC2278385

[b36] Parikh C. R. McCall D. Engelman C. Schrier R. W. 2002 Congenital renal agenesis: case‐control analysis of birth characteristics. Am. J. Kidney Dis.; 39:689-694 11920333 10.1053/ajkd.2002.31982

[b37] Persson A. E. Gushwa L. C. Blantz R. C. 1984 Feedback pressure‐flow responses in normal and angiotensin‐prostaglandin‐blocked rats. Am. J. Physiol. Renal Physiol.; 247:F925-F931 10.1152/ajprenal.1984.247.6.F925 6507631

[b38] Pollock C. A. Bostrom T. E. Dyne M. Gyory A. Z. Field M. J. 1992 Tubular sodium handling and tubuloglomerular feedback in compensatory renal hypertrophy. Pflügers Arch.; 420:159-166 1620575 10.1007/BF00374985

[b39] Reckelhoff J. F. 2008 Sex and sex steroids in cardiovascular‐renal physiology and pathophysiology. Gend. Med.; 5Suppl. A:S1-S2 18395674 10.1016/j.genm.2008.03.001

[b40] Rueda‐Clausen C. Morton J. Davidge S. T. 2011 The early origins of cardiovascular health and disease: who, when, and how. Semin. Reprod. Med.; 29:197-210 21710396 10.1055/s-0031-1275520

[b41] Schnermann J. Briggs J. 2008 Tubuloglomerular feedback: mechanistic insights from gene‐manipulated mice. Kidney Int.; 74:418-426 18418352 10.1038/ki.2008.145 PMC2562927

[b42] Schnermann J. Traynor T. Yang T. Arend L. Huang Y. G. Smart A. 1998 Tubuloglomerular feedback: new concepts and developments. Kidney Int.; 54:S40-S45 10.1046/j.1523-1755.1998.06708.x 9736251

[b43] Schreuder M. F. Langemeijer M. E. Bökenkamp A. Delemarre‐Van de Waal H. A. van Wijk J. A. 2008 Hypertension and microalbuminuria in children with congenital solitary kidneys. J. Paediatr. Child Health; 44:363-368 18476930 10.1111/j.1440-1754.2008.01315.x

[b44] Singh R. R. Denton K. M. Bertram J. F. Jefferies A. J. Head G. A. Lombardo P. 2009 Development of cardiovascular disease due to renal insufficiency in male sheep following fetal unilateral nephrectomy. J. Hypertens.; 27:386-396 19155792 10.1097/HJH.0b013e32831bc778

[b45] Singh R. R. Denton K. M. Bertram J. F. Jefferies A. J. Moritz K. M. 2010 Reduced nephron endowment due to fetal uninephrectomy impairs renal sodium handling in male sheep. Clin. Sci.; 118:669-680 20067444 10.1042/CS20090479

[b46] Singh R. R. Jefferies A. J. Lankadeva Y. R. Lombardo P. Schneider‐Kolsky M. Hilliard L. 2012a Increased cardiovascular and renal risk is associated with low nephron endowment in aged females: an ovine model of fetal unilateral nephrectomy. PLoS One; 7:e42400 22879965 10.1371/journal.pone.0042400 PMC3411741

[b47] Singh R. R. Moritz K. M. Bertram J. F. Denton K. M. 2012b Renal responses to furosemide are significantly attenuated in male sheep at 6 months of age following fetal uninephrectomy. Am. J. Physiol. Regul. Integr. Comp. Physiol.; 302:R868-R875 22319047 10.1152/ajpregu.00579.2011

[b48] Singh R. R. Lankadeva Y. R. Denton K. M. Moritz K. M. 2013 Improvement in renal hemodynamics following combined angiotensin II infusion and AT1R blockade in aged female sheep following fetal unilateral nephrectomy. PLoS One; 8:e68036 23840884 10.1371/journal.pone.0068036 PMC3698080

[b49] Thomson S. C. Blantz R. C. Vallon V. 1996 Increased tubular flow induces resetting of tubuloglomerular feedback in euvolemic rats. Am. J. Physiol. Renal Physiol.; 270:F461-F468 10.1152/ajprenal.1996.270.3.F461 8780249

[b50] Tucker B. J. Blantz R. C. 1984 Effect of furosemide administration on glomerular and tubular dynamics in the rat. Kidney Int.; 26:112-121 6503131 10.1038/ki.1984.144

[b51] Vallon V. 2003 Tubuloglomerular feedback in the kidney: insights from gene‐targeted mice. Pflügers Arch.; 445:470-476 12548391 10.1007/s00424-002-0952-4

[b52] Vize P. D. Seufert D. W. Carroll T. J. Wallingford J. B. 1997 Model systems for the study of kidney development: use of the pronephros in the analysis of organ induction and patterning. Dev. Biol.; 188:189-204 9268568 10.1006/dbio.1997.8629

[b53] Westland R. Kurvers R. A. J. van Wijk J. A. E. Schreuder M. F. 2013 Risk factors for renal injury in children with a solitary functioning kidney. Pediatrics; 131:e478-e485 23319536 10.1542/peds.2012-2088

[b54] Woods L. Weeks D. Rasch R. 2004 Programming of adult blood pressure by maternal protein restriction: role of nephrogenesis. Kidney Int.; 65:1339-1348 15086473 10.1111/j.1523-1755.2004.00511.x

[b55] Woods L. L. Ingelfinger J. R. Rasch R. 2005 Modest maternal protein restriction fails to program adult hypertension in female rats. Am. J. Physiol. Regul. Integr. Comp. Physiol.; 289:R1131-R1136 15961538 10.1152/ajpregu.00037.2003

[b56] Wright F. S. Schnermann J. 1974 Interference with feedback control of glomerular filtration rate by furosemide, triflocin, and cyanide. J. Clin. Invest.; 53:1695-1708 4830232 10.1172/JCI107721 PMC302666

